# GPX4 is a potential diagnostic and therapeutic biomarker associated with diffuse large B lymphoma cell proliferation and B cell immune infiltration

**DOI:** 10.1016/j.heliyon.2024.e24857

**Published:** 2024-01-26

**Authors:** Can Chen, TongYu Li, Yiwei Li, Zhenzhen Chen, Pengfei Shi, Yun Li, Shenxian Qian

**Affiliations:** a Department of Hematology, Affiliated Hangzhou First People's Hospital, West Lake University, School of Medicine, Hangzhou, China; b Department of Hematology, Ningbo First Hospital, Ningbo, China; c Team of Neonatal & Infant Development, Health and Nutrition, NDHN, School of Biology and Pharmaceutical Engineering, Wuhan Polytechnic University, Wuhan, China; d Kindstar Global Precision Medicine Institute, Wuhan, China; e Department of Scientific Research Project, Wuhan Kindstar Medical Laboratory Co., Ltd., Wuhan, China

**Keywords:** DLBCL, GPX4, Single cells, B cell immune, Biomarker

## Abstract

At present, GPX4's role in the occurrence and development of diffuse large B lymphoma (DLBCL) is rarely reported. This study's purpose is to explore GPX4's significance in the diagnosis, treatment, and pathological mechanisms of DLBCL. The TIMER 2.0, GEPIA, and GEO databases were used to analyze GPX4's expression levels in DLBCL tissue, peripheral blood, and single cells, and evaluate its potential performance as a therapeutic and diagnostic marker. Cell experiments validate GPX4's role in DLBCL cells. And revealed the potential mechanism of GPX4's action from three aspects: immunity, pathogenic gene expression, and protein interaction. The results indicate that GPX4 can be used as a biomarker for treatment and diagnosis (FC > 1.5, P < 0.05, AUC>0.8, KM-P value < 0.05). In single cell data, GPX4 also showed high expression in immune cells. Besides, cell experiments have confirmed that GPX4's high expression can inhibit DLBCL cells' proliferation. Meanwhile, we found a negative correlation between GPX4 and the 16 core DLBCL's pathogenic genes, and a significant negative correlation with immune B cell infiltration. In summary, GPX4 can serve as a potential therapeutic and diagnostic marker for DLBCL. GPX4's high expression can lead to a good prognosis in DLBCL patients, which may be related to its inhibition of cancer cell proliferation, high expression of key pathogenic genes, and infiltration of immune B cells.

## 1 Introduction

DLBCL is the most common hematological malignancy in the world, and its incidence rate has risen rapidly in the last 20 years [1]. DLBCL is a group of tumors with biological heterogeneousness, and its clinical prognosis is quite different. DLBCL can be divided into ABC groups and GCB according to gene expression. Patients with GCB gene expression had longer survival than those with ABC. Although most lymphoma patients have been treated with radical surgery successfully, a certain proportion of patients show high aggressiveness by staying unresponsive to treatment, and some patients may relapse after treatment [2]. It therefore is essential to further understand and clarify DLBCL's molecular mechanism to provide better clinical decision-making and effective treatment strategies.

More and more evidence shows that ferroptosis disorder plays an important role in the development, recurrence and occurrence of multiple cancers, including DLBCL [3,4]. Besides, ferroptosis's imbalance is considered a valuable biomarker for the prognosis and diagnosis of DLBCL [5]. One of the most studied ferroptosis genes is TFRC and GPX4. A large number of studies have found that GPX4 can be used as one of the indicators to judge cell ferroptosis [6,7].

It was found that overexpression or knockdown of GPX4 could regulate 12 ferroptosis inducers' lethality to cells. GPX4 has been therefore confirmed as a key regulatory factor of ferroptosis [8]. GPX4's specific mechanism is to use its catalytic activity to weaken lipid peroxide's toxicity and maintain membrane lipid bilayer's stability. Cells with GPX4 expression down regulated are more sensitive to ferroptosis, while those with GPX4 expression up regulated inhibit ferroptosis. GSH reduces active nitrogen and ROS under GPX's action [9]. RSL3 ((1S, 3R) -- RSL3) is an inhibitor of GPX4. RSL3 covalently that is bound with GPX4 inactivates GPX4, leading to intracellular peroxides' accumulation, leading to ferroptosis [10]. As a synergistic factor of GPX4 in catalyzing peroxide's conversion to alcohol, glutathione deficiency will lead to cysteine deficiency, which will inactivate GPX4 directly and cause ferroptosis. The research results show that targeting GPX4 directly can induce cancer cells' ferroptosis more effectively [11]. To this end, researchers have found a lot of GPX4 inhibitors, such as RSL3, ML162, DPI compounds, FIN56 and FINO2. Poor pharmacokinetic properties, however, limit most current GPX4 inhibitors' clinical application [12].

Research shows that compared with normal tissues, GPX4's expression level in tumor tissues is significantly higher, such as colon adenocarcinoma, renal chromophobe cell, renal clear cell carcinoma, lung adenocarcinoma, prostate cancer, rectal adenocarcinoma, thyroid cancer and endometrial cancer. It is speculated that GPX4 may be an oncogene [13,14]. Besides, some researchers believe that GPX4 is closely related to cancer patients' poor prognosis. As an important cell signal molecule, ROS is considered one of the important characteristics of development and tumorigenesis. ROS's accumulation is related to tumor stem's production cells and the process of mesenchymal transformation of tumor epithelial cells. Tumors' EMT process is known as the “tumor proliferation's culprit”. GPX4 has been proved to affect tumor EMT by regulating ROS molecules [15].

Currently, GPX4 as the core regulator of ferroptosis, It has been considered a “star molecule” in cell ferroptosis's study, which has attracted a lot of attention [16]. GPX4's abnormal expression plays an important role in a lot of cancers' tumorigenesis, such as breast cancer, colorectal cancer, thyroid cancer and hepatocellular carcinoma [17]. Related studies have found that GPX4 is crucial in regulating DLBCL's growth and plays a role as an oncogene [18]. The overexpression that is reported in the literature is considered to be a poor prognostic predictor of DLBCL. GPX4 overexpression's mechanism, however, is still unknown [19]. Based on these limited studies, GPX4's role in diagnosis, prognosis and mechanism in DLBCL is still unclear, so further GPX4's role in DLBCL is needed. In this study, we therefore used comprehensive bioinformatics methods to evaluate GPX4's differential expression in its correlation and pan carcinoma with prognosis, immune cell infiltration and DLBCL diagnosis.

## 2 Materials and methods

### 2.1 Type of the study

This study is a molecular genetics research mainly focussing on the expression of gene (GPX4) in DLBCL, the correlation between GPX4 and DLBCL pathology and immunity, and verifies GPX4's functional role in two types of DLBCL cells.

### 2.2 Microarray data acquisition and processing

The gene expression microarray dataset for diagnostic performance evaluation and GPX4 expression level includes GSE56315, GSE83632 and TCGA. The dataset used to evaluate GPX4 as a potential prognostic marker after DLBCL treatment includes GSE181063 and TCGA、GSE10846, and the GEO dataset can be downloaded from the Gene Expression Comprehensive Database (GEO) (https://www.ncbi.nlm.nih.gov/geo/). The GSE56315 dataset was obtained from the GPL570 platform, including 33 matched normal samples and 55 DLBCL samples. The GSE dataset was obtained from the GPL5175 platform, including peripheral blood samples from 87 healthy donors and 76 DLBCL patients. The GSE10846 dataset was obtained from the GPL570 platform, including clinical samples of 181 CHOP that was treated patients and 233 Rituximab CHOP treated patients. The GSE181063 dataset was obtained from the GPL14951 platform, including 1311 confirmed DLBCL formalin fixed paraffin embedded (FFPE) samples. Single cell data from GSE175510. We used the R 4.2.1 tool to evaluate prognostic risk assessment and gene expression levels by analyzing raw data from microarrays [20].

### 2.3 Differential expression of GPX4 in pan-cancers

To confirm that GPX4 is a potential oncogene, we analyzed GPX4's pan oncogene expression level through the TCGA database. Gene Expression Profiling Interaction Analysis (GEPIA, http://gepia.cancer-pku.cn/index/html) It can be used to evaluate the RNA that expression data of 8587 normal TCGA samples and 9736 tumor samples, as well as the genotype tissue expression (GTEx) database [21,22]. When obtaining GPX4's gene expression profile, ANOVA method was used to compare with the following thresholds: | log2FC | cutof = 1, LogScale = log2 (TPM+1) and q value cutof = 0.01 [23].

### 2.4 GPX4 is a potential biomarker for DLBCL tissue、peripheral blood and scRNA-seq

To evaluate GPX4's diagnostic value in DLBCL, we first used DLBCL tissue samples from the GSE56315 datasets and GEPIA, and evaluated GPX4's diagnostic value in both tissue samples using ROC curves. GEPIA obtained data from TCGA for gene expression analysis and performed ROC curves. GEO data GSE56315 was used to validate ROC curve diagnostic performance and GPX4 gene expression data in DLBCL tissue samples. The inclusion criteria for DLBCL tissue samples are: (a) samples diagnosed with DLBCL, (b) samples with RNA sequence data, and (c) samples containing complete clinical information. The exclusion criteria for DLBCL samples are as follows: (a) normal tissue samples, (b) metastatic tissue samples, and (c) samples lacking expression data and complete clinical information [24]. Due to the convenience and ease of using blood for disease diagnosis in clinical practice, we evaluated the diagnostic performance and expression of GPX4 in DLBCL's peripheral blood using the GSE83632 dataset. In order to further evaluate GPX4's expression at DLBCL single cell transcriptome's level, we evaluated GPX4's expression level at DLBCL single cell transcription's level through single cell data GSE175510 [22].

### 2.5 Prognostic analysis of GPX4 in DLBCL

In order to evaluate GPX4's prognostic role in DLBCL, the prognosis of DLBCL patients after treatment was evaluated by analyzing GPX4's high and low expression with KM curve, and then verified in TCGA dataset to evaluate GPX4's potential ability as a prognostic marker of DLBCL [23].

### 2.6 Cells, reagents, and instruments

OCI-LY8 cells are sourced from the cell bank of Ningbo First Hospital in Zhejiang Province. OCI-LY1 cells are sourced from the cell bank of Hangzhou First Hospital in Zhejiang Province. DMEM culture medium (Gibco, USA); Instrument CO2 constant temperature incubator (Shanghai Thermo Company); Ultra clean workbench (Suzhou Sujing Group Antai Company); Gilson, Ependorf, IMDM, FBS, Solarbio, PI staining solution, PCR instrument purchased from ABI Company in the United States; The flow cytometry was purchased from Thermo Corporation in the United States [25].

### 2.7 Cell overexpression experiment

OCI-LY8 cells were inoculated in DMEM medium that contained 10 % fetal bovine serum (containing 100 mg/mL streptomycin and 100 U/mL penicillin), and cultured at 37 °C in 5 % CO2 incubator. When the adherent parietal cell grows into a compact monolayer, it is subcultured. Partial stably growing DLBCL cancer cells were divided into two groups randomly: the empty control group and the GPX4 overexpression group. The nonsense sequence GPX4 plasmid vector and The GPX4 overexpression plasmid vector were transfected into DLBCL cancer cells, respectively [26].

### 2.8 Flow cytometry detection

Absorb and discard the culture medium, clean the cells with 2 ml PBS, add 1 ml trypsin, digest in a 37 °C incubator for 1 min, add the culture medium to terminate digestion, collect the cell suspension, centrifuge at 1000 rpm for 5 min, and discard the supernatant; Add 5 ml of pre that is cooled 70 % ethanol, mix well while adding, and fix overnight at 4 °C; Cell staining: collect cells by centrifugation, wash cells twice with 5 mL PBS, centrifuge at 1000 rpm for 5 min, and discard the supernatant. Add 500 μL of transparent liquid (100 μ G/mL RNase A, 0.2 % Triton X-100) incubated at 37 °C for 30 min. Wash cells twice with PBS's 5 mL, 50 μ G/mL ethidium bromide (PI) staining for 10 min. Pass through a 300 mesh sieve, centrifuge at 1000 rpm for 5 min, and discard the supernatant. Add 5 ml PBS to wash excess PI off, use 200 μ PBS resuspended cells and detected them on the machine [27].

### 2.9 Construction of cell model for GPX4 interference

SiRNA is a chemically synthesized small molecule serving as an important intermediate for gene that silences and sequence specific RNA degradation. It has special structural features such as a 5 ′end phosphate group and a 3′ end hydroxyl group, with two free bases at the 3 ′end of each of its two chains. It degrades mRNA through specific complementary binding with the target mRNA. We constructed three siRNAs (GPX4 siRNA1, GPX4 siRNA2, and GPX4 siRNA3) based on the GPX4 sequence. After transfection into Oci-LY1 cells, these three siRNAs' interference effect on GPX4 was verified through qPCR. A random sequence was used as the control group (GPX4 NC), and GPX4 interference's cell model was constructed by selecting one of the three siRNAs with the significant interference effect and best interference effect [28].

### 2.10 Cell cycle experiment

Firstly, discard the culture medium, wash the cells with 2 ml PBS, add 1 ml trypsin, digest in a 37 °C incubator for 1 min, terminate digestion with the medium, collect the cell suspension, centrifuge at 1000 rpm for 5 min, discard the supernatant, add 5 ml of pre that is cooled 70 % ethanol, mix well while adding, and fix overnight at 4 °C. Then collect the cells by centrifugation, wash them twice with PBS's 5 mL, centrifuge at 1000 rpm for 5 min, discard the supernatant, and add 500 μL of permeable solution (100 μ G/mL RNase A, 0.2 % Triton X-100) incubated at 37 °C for 30 min. Subsequently wash the cells twice with PBS's 5 mL, 50 μ Dye with g/mL ethidium bromide (PI) for 10 min, pass through a 300 mesh sieve, centrifuge at 1000 rpm for 5 min, and discard the supernatant. Finally, add 5 ml PBS to wash excess PI off and use 200 μ After PBS cells' resuspension, flow cytometry detection was performed [28].

### 2.11 Cell apoptosis experiment

First, collect cells (1 × 106 pieces/time), wash with cold PBS, and then use 1 ml 1 × that Binds Buffer suspension cells, 300 × Centrifuge for 10 min and discard the supernatant. Using 1 ml 1 × The Binding Buffer resuspended cells to achieve a cell density of 1 × 106 pieces/ml. Then add 100 to each tube μ L cells (1 × 105) and 5 μ L Annexin V-FITC, gently mix at dark conditions and room temperature for 10 min, then add 5 μ L PI, room temperature, dark, incubate for 5 min. Finally, add PBS to 500 μ L. Gently mix and detect within 1 h using a flow cytometry [29].

### 2.12 Correlation analysis of GPX4 and DLBCL key pathogenic genes

We used TCGA data to analyze the differential genes (P < 0.01 and Log2>4) that were up-regulated in DLBCL ranking, and then used R soft 4.2.1 to analyze the GEO dataset GSE56315 to verify TCGA dataset's results. Utilize STRING database (https://string-db.org/) Obtain differential genes' network diagram in DLBCL, and obtain key functional gene modules through the MCODE add-in in the Cytoscape software. Spearman correlation was then used to analyze the correlation between DLBCL key MCODE genes and GPX4 [30].

### 2.13 Analysis of GPX4 related protein interaction network

We use GeneMANIA (https://genemania.org/) A PPI network that was centered on GPX4 was constructed, which included the correlation data of genetic interactions and protein, pathways, co expression, co localization and protein domain similarity. Then GO function enrichment and KEGG pathway analysis were carried out for the gene that was centered on GPX4 that was constructed by GeneMANIA [31].

### 2.14 Correlation analysis between GPX4 and immune cell infiltration

The TIMER database is a comprehensive resource that can be used to assess multiple cancer types' immune effects systematically. We used TIMER to analyze the correlation between CPX4 and key related genes, their copy numbers of CPX4 and key related genes, and a large number of tumor infiltrating immune cells (CD4+T cells, CD8+T cells, B cells, dendritic cells, neutrophils and macrophages) in DLBCL [22].

### 2.15 Statistical analysis

As mentioned above, most statistical analyses of differential gene expression were performed using R 4.2.1 and online databases. Student t-test was used for comparison between the two groups. Kaplan Meier method was used for log rank test and survival analysis. For all results, p < 0.05 was considered statistically to be significant [21].

## 3 Result

### 3.1 Expression of GPX4 in pan-cancers

**Fig. 1:**
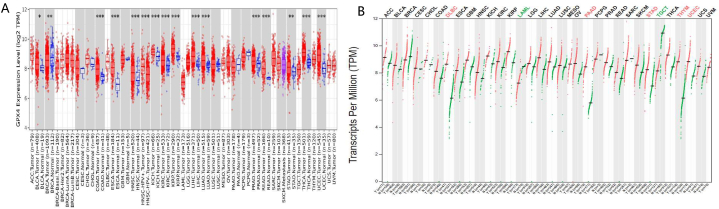
The expression level of GPX4 in different cancers. TIMER was used to evaluate the expression level of GPX4 in different tumor types in TCGA data. （*P < 0.05，**P < 0.01，***P < 0.001）。 The expression of GPX4 was significantly up-regulated in BLCA, COAD, ESCA, HNSC, KICH, KIRC, KIRP, LIHC, LUAD, PRAD, READ, STAD, THCA and UCEC. However, GPX4 expression was significantly down regulated in BRCA (A). The difference of GPX4 expression level in different tumors and normal tissues of TCGA was detected by GEPIA in GTEx database. Red represents tumor tissue, green represents normal tissue. GPX4 expression was significantly increased in DLBC, PAAD, STAD, THYM and UCEC, however, GPX4 expression was significantly decreased in LAML and TGCT (B). (For interpretation of the references to color in this figure legend, the reader is referred to the Web version of this article.)

GEPIA online databases were used to analyze GPX4's expression level in different types of normal tissues and human cancer. We used GEPIA2 database and TIMER 2.0 database, which integrated gene expression data from GTEx and TCGA to determine GPX4's expression level in 337 normal tissues and 48 human tumors (Fig. 1A and B). According to this result, compared with adjacent normal tissues, GPX4's expression in DLBC, PAAD, STAD, THYM and UCEC was significantly increased, however, GPX4's expression was significantly decreased in TGCT and LAML (Fig. 1B). In TCGA database, GPX4's expression in a lot of cancers has changed significantly, and most of them are significantly up-regulated. GPX4 may therefore be a potential oncogene.

### 3.2 GPX4 is a potential biomarker for DLBCL tissue、peripheral blood and scRNA-seq

**Fig. 2:**
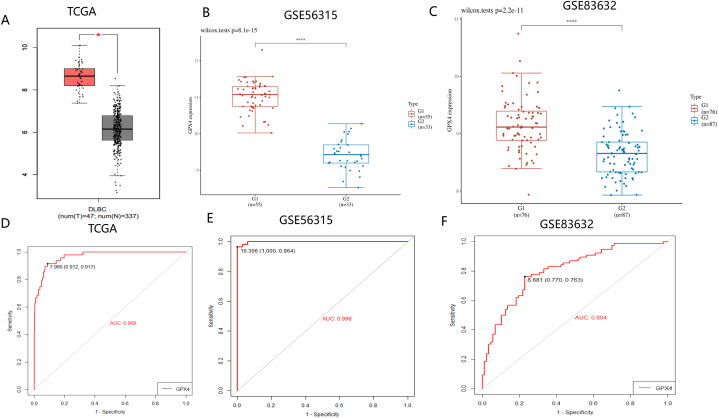
Up regulation of GPX4 expression in DLBCL and correlation between diagnostic performance evaluation. By analyzing the expression of GPX4 in normal and tumor tissues and peripheral blood using TCGA RNA seq data, GPX4 expression was upregulated in tumor tissues (red represents DLBCL like, black or blue represents control group samples) (A–B) and peripheral blood (C). TCGA data and GSE56315 data were used to determine the expression level and diagnostic performance of GPX4 in DLBCL. The expression of GPX4 is significantly upregulated in DLBCL tissue, with AUC>0.95, indicating excellent diagnostic performance (D–E), as well as excellent diagnostic performance in peripheral blood (F) with AUC>0.8. (For interpretation of the references to color in this figure legend, the reader is referred to the Web version of this article.)

We further analyzed GPX4's gene expression level in DLBCL using the on-line database GEPIA. According to this result, GPX4 is upregulated in cancer tissue compared to normal DLBCL tissue. In the TCGA dataset, we found a clear stratification between normal tissue samples and DLBCL tissue, indicating that GPX4 may be a potential diagnostic marker for DLBCL (Fig. 2A). We therefore validated GPX4's differential expression in GSE56315 (Fig. 2B) and further analyzed it in peripheral blood samples' GSE83632 dataset. We found that GPX4 was significantly upregulated in DLBCL peripheral blood samples (Fig. 2C). Based on the scRNA seqTISCH database, independent dataset of DLBCL was subjected to single cell sequencing to explore the correlation between GPX4 expression level and immune cell distribution at the single cell level. GPX4 expression's high levels were found in all four cells, especially in CD8T cells, Mono/Macro, and Malignant cells (Figs. 1A–C). To evaluate GPX4's potential as a diagnostic marker for DLBCL, the ROC curve In DLBCL tissue was first analyzed. GSE56315 and TCGA show that GPX4 has good discriminative ability between normal tissue samples and cancer samples (AUC>0.98) (Fig. 2D and E). In order to evaluate GPX4's clinical application ability (blood samples can alleviate patient pain and facilitate sampling in clinical practice), we evaluated GPX4's diagnostic performance in DLBCL's peripheral blood (AUC>0.8). GPX4 therefore is a good potential diagnostic marker for DLBCL (Fig. 2F).

### 3.3 Prognostic analysis of GPX4 in DLBCL

**Fig. 3:**
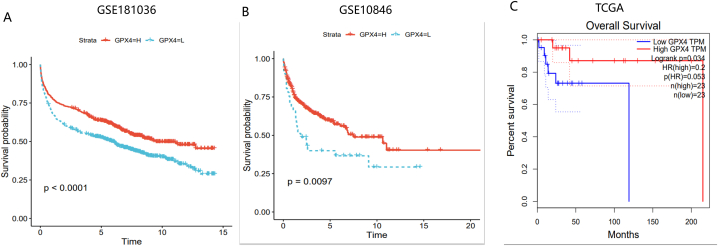
GPX4 prognosis assessment in DLBCL. The prognosis performance of GPX4 in DLBCL was evaluated by using TCGA database, GSE181063 and GSE10846. The prognosis difference between high and low expression of GPX4 in DLBCL was significant in the three data sets (*P < 0.05) (A–C).

In order to evaluate GPX4's prognostic role in DLBCL, KM curve also showed that there was a significant difference between patients' survival curves with low and high expression of GPX4, which was verified in TCGA data set (P < 0.05) (Fig. 3A–C). GPX4's high expression therefore leads to a good prognosis in DLBCL patients. Compared with TP53 wild-type patients, GPX4's expression level in TP53 mutant patients was significantly increased and the KM curve showed that TP53 mutant patients had a better prognosis (Fig. 2A). GPX4's high expression level therefore promoted a good prognosis for patients.

### 3.4 The effect of overexpression of GPX4 on the proliferation of DLBCL cancer cells

Flow cytometry cycle test results showed that the DLBCL cancer cells transfected with GPX4 gene overexpression decreased in G1 phase, rose in S phase, and decreased in G2 phase, indicating that GPX4 gene overexpression led to DLBCL cancer cells' accelerated synthesis in G1 phase but was blocked in S phase, thus affecting DLBCL cancer cells' proliferation (appendix Fig. 3A). GPX4 gene's overexpression therefore inhibited DLBCL cancer cells' proliferation.

### 3.5 Cell cycle experiment

**Fig. 4:**
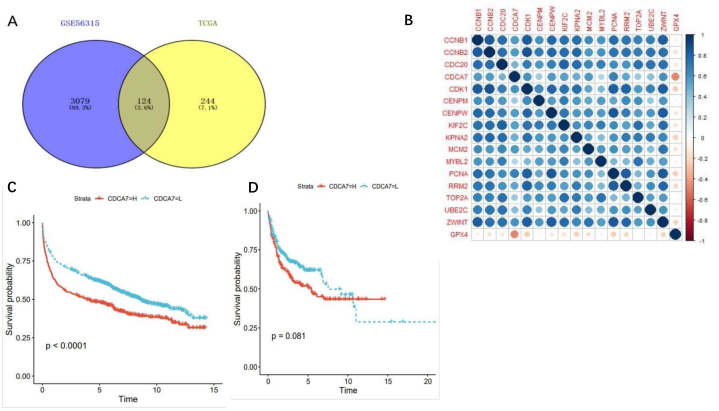
Correlation analysis of GPX4 and DLBCL key genes We analyzed the differential genes (Log2>4 and P < 0.01) that were up-regulated in DLBCL ranking by TCGA data, and then verified them by GSE56315 (A). A total of 124 differential genes have been verified. Utilize STRING database（ https://string-db.org/）The network relationship diagram of 124 differential genes in DLBCL was obtained, and the key functional gene modules were obtained through the MCODE plug-in in the Cytoscape software. We took the module with the highest total score (Score>14), and a total of 16 key genes were obtained and spearman correlation analysis was used to analyze the correlation between GPX4 and the six key MCODE genes of DLBCL (B). The results showed that all these genes had a negative correlation with GPX4, indicating that the upregulation of the key genes of this module promoted the occurrence and development of DLBCL, and there was a significant negative correlation between GPX4 and CDCA7. Then we conducted a prognostic analysis of CDCA7 and found that CDCA7 was a risk factor (Fig. C–D).

**Fig. 5:**
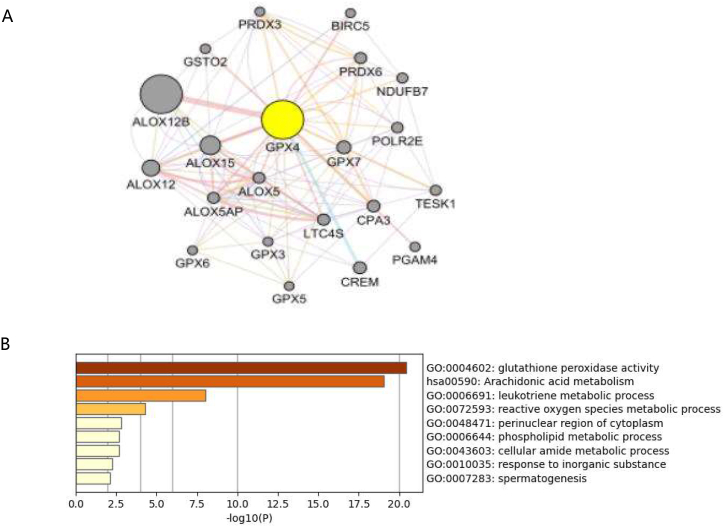
Co-expression network analysis of GPX4 in DLBCL GPX4 prognosis assessment in DLBCL. The A GPX4 centered PPI network was constructed using GeneMANIA (A), and GO function enrichment and KEGG pathway analysis were conducted for the GPX4 centered genes constructed using GeneMANIA (B).

The qPCR results showed that compared with the control group, the cells transfected with OCI-LY1+GPX4 -siRNA3 had the most significant interference effect on GPX4 (Fig. 4A). The cells transfected with OCI-LY1+GPX4 -siRNA3 were therefore selected as the cell model for GPX4 interference and named OCI-LY1+GPX4-siRNA-485. The cell cycle results showed that Compared with group OCI-LY1+NC-siRNA cells, group OCI-LY1+GPX4-siRNA-485 showed a decrease in G1 phase cells, no significant changes in S phase cells, and an rise in G2 phase cells (Figs. 5A–B). GPX4's low expression therefore promoted the DLBCL cell cycle's progression significantly.

### 3.6 Cell apoptosis experiment

**Fig. 6:**
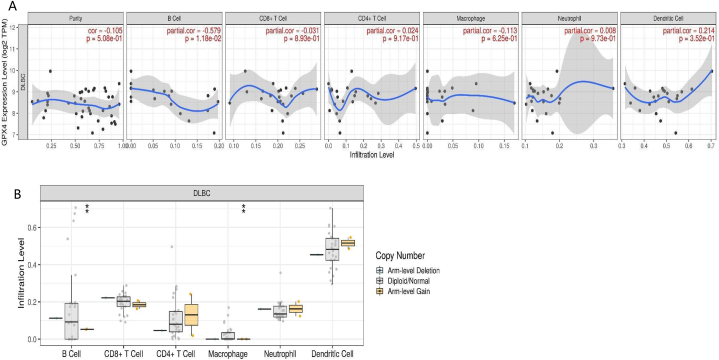
Correlation between GPX4 in DLBCL and immune cell influence level. The expression level of GPX4 is correlated with the influence level of different immune cells in DLBCL (A). Correlation of the influence of GPX4 copy number change on immune cells in DLBCL (B).

Cell apoptosis' results showed that the OCI-LY1+GPX4-siRNA-485 group showed a significant decrease in the apoptosis rate compared to the OCI-LY1+NC-siRNA group cells (Figs. 6A–B). GPX4's low expression therefore inhibited DLBCL cells' apoptosis significantly.

### 3.7 Correlation analysis of GPX4 and DLBCL key pathogenic genes

We analyzed the differential genes (Log2>4 and P < 0.01) that were up-regulated in DLBCL ranking by TCGA data, and then verified them by GSE56315, A total of 124 differential genes have been verified (Fig. 4A).

Utilize STRING database (https://string-db.org/) the network relationship diagram of 124 differential genes in DLBCL was obtained, and the key functional gene modules were obtained through the MCODE add-in in the Cytoscape software. We took the module with the highest total score (Score>14), and a total of 16 key MCODE genes were obtained and spearman correlation analysis was used to analyze the correlation between GPX4 and the 16 key MCODE genes of DLBCL (Fig. 4B), the results showed that all these genes had a negative correlation with GPX4, indicating that the upregulation of this module's key genes promoted the development and occurrence of DLBCL, and there was a significant negative correlation between CDCA7 and GPX4. We then conducted a prognostic analysis of CDCA7 and found that CDCA7 was a risk factor (Fig. 4C and D), CDCA7 gene encodes 371 amino acid proteins, which are abnormally expressed in various tumor tissues. At present, there are no reports on CDCA7's role in DLBCL. However, literature has reported that CDCA7 plays a crucial role in the pathogenesis of lymphoma [32]. Our results demonstrate that CDCA7 is DLBCL's core pathogenic gene, and CDCA7's high expression leads to poor prognosis in DLBCL patients. GPX4 may therefore reduce 16 key pathogenic genes' expression in DLBCL patients, reducing CDCA7's expression significantly, and promoting a good prognosis for patients.

### 3.8 Analysis of GPX4 related protein interaction network

GeneMANIA was used to build a PPI network of 21 genes that were centered on GPX4 (Fig. 5A). GO function enrichment and KEGG pathway analysis were carried out for these 21 genes. Significantly rich GO terms include Glutathione peroxidase activity, Leukotriene metabolic process, Reactive oxygen species metabolic process, while the significantly rich KEGG pathway is Arachidonic acid metabolism, which has been reported to be closely related to lymphoma's immune regulation in recent years (Fig. 5B). These results suggest that GPX4 may be an important regulator of DLBCL progression by participating in important metabolic pathways that are related to immune cell infiltration and lymphoma.

### 3.9 Correlation between GPX4 and immune cell infiltration

Tumor microenvironment has been proved to play an important role in tumorigenesis. We used TIMER to determine whether the expression of GPX4 and GPX4 copy number changes in DLBCL is related to immune cell infiltration. We found that GPX4 is significantly negatively related to immune B cell infiltration (Fig. 6A), The change of the copy number of GPX4 was correlated with the infiltration of immune macrophages and immune B cells significantly (Fig. 6B). The immune interaction at the expression level therefore is mainly related to immune B cells' infiltration, while the immune interaction at the copy number variation level is mainly related to immune macrophages.

## 4 Discuss

Immunotherapy has shown therapeutic effects on a variety of malignant tumors in recent years. One of the key cell types in the innate immune response is macrophages. GPX4 is a key protein inhibiting ferroptosis. Knocking GPX4 gene out in MZ B cells and B1 will trigger cell ferroptosis by inducing lipid peroxidation, thus affecting the immune response of B cells [33]; Studies have shown that RSL3, as an inhibitor of GPX4, is used to induce cell ferroptosis's occurrence widely. Because of the low content of iNOS in M2 macrophages, nitric oxide free's production radicals is less, and lipid peroxidation's inhibition is less, RSL3 can induce M2 macrophages to express ferroptosis [34]; Our study found that GPX4 was significantly negatively correlated with immune B cell infiltration (Fig. 5A), and GPX4 copy number's change was correlated with immune macrophage infiltration and immune B cell significantly. In view of the relationship between GPX4 and macrophages and immune B cells, a lot of studies are analyzing GPX4's role in tumor immunotherapy.

At present, there are a lot of inhibitors that are related to GPX4. Such as RSL3, ML162, DPI compounds, FIN56 and FINO2. Poor pharmacokinetic properties, however, limit most current GPX4 inhibitors' clinical application. For instance, RSL3, as an inhibitor of GPX4, is used to induce cell ferroptosis widely. The content of nitric oxide free radicals in M1 cells is higher, thereby inhibiting lipid peroxidation;, because the content of iNOS in pro-inflammatory M1 macrophages is higher than that in anti-inflammatory M2 macrophages On the contrary, M2 macrophages produce less nitric oxide free radicals due to lower iNOS content, and have less inhibition on lipid peroxidation. RSL3 can therefore not induce M1 macrophages to die of iron, but can induce M2 macrophages to die of iron [35]. These studies revealed GPX4's mechanism in DLBCL from a horizontal perspective, while this study explored the correlation and influence of GPX4 expression on DLBCL from a vertical perspective.

GPX4 shows a strong therapeutic and diagnostic ability in DLBCL, AUC = 0.9 or above, which can be used as a potential diagnostic marker of DLBCL. Besides, GPX4 has a significant difference between patients with high patients and expression with low expression after DLBCL treatment. GPX4 has a good prognostic evaluation ability in all three data sets, so GPX4 can also be used as an independent factor and a potential prognostic marker for prognosis evaluation after DLBCL treatment.

This study found a significant correlation between CDCA7 and GPX4. CDCA7 gene encodes 371 amino acid proteins, which are abnormally expressed in various tumor tissues [36]. At present, CDCA7's role in DLBCL has not been reported. Our results show that CDCA7 is significantly up-regulated in multiple DLBCL data sets, and is a significant prognostic factor for poor DLBCL in 1311 samples. CDCA7 may therefore be a key pathogenesis gene and a key therapeutic and diagnostic molecule for DLBCL. Some studies have shown that tumors' formation involves a large number of abilities that are acquired in the process of developing from normal cells to malignant cells, including unlimited proliferation (immortalization) and non anchored growth, which are closely related to tumors' occurrence [37]. Compared with the tissues and control cell lines, the CDCA7 protein was up-regulated in human tumor biopsy specimens and Burkitt lymphoma cell lines, respectively. CDCA7 levels were also significantly elevated in a lot of T and B lymphoid tumor cell lines. Although CDCA7 is unnecessary for anchoring normal fibroblasts' dependent growth or non malignant lymphocytes, it is necessary but not adequate for anchoring independent growth of lymphoid tumors and lymphoid tumor cells [38]. These data suggest that treatment aimed at inhibiting the expression or CDCA7's function may reduce lymphomas' growth significantly. GPX4's high expression may therefore inhibit CDCA7's expression, thus preventing the development, poor prognosis and occurrence of DLBCL.

Besides, key genes' functional pathways were analyzed. The results that were showed that DLBCL related genes to were significantly enriched in the pathways that were related to ferroptosis, including Glutathione peroxidase activity, Leukotriene metabolic process, Reactive oxygen species metabolic process, and Arachidonic acid metabolism. These evidences indicate that ferroptosis is closely related to DLBCL. However, whether GPX4's expression can promote the development and occurrence of DLBCL by influencing ferroptosis related pathways is not still conclusive. This study proposes this hypothesis here, but the specific mechanism still needs further experimental verification.

In this study, we analyzed the prognosis and expression of GPX4 in DLBCL, and analyzed GPX4's functional pathway and the correlation of immune cell infiltration. We found that there were differences in the prognosis and expression of GPX4 in DLBCL. The difference in prognosis and GPX4 expression may reflect the abnormal immune status of DLBCL, which may be related to DLBCL's pathogenesis. Next, we will verify GPX4's functional mechanism in its subtypes and DLBCL through experiments to provide the treatment and diagnosis of DLBCL a theoretical basis.

## 5 Conclusion

This article's main purpose is to explore the clinical therapeutic and diagnostic effects of GPX4, as well as the mechanism by which GPX4's high expression leads to good prognosis in patients. Firstly, we explored GPX4's diagnostic role in clinical practice. We analyzed GPX4's expression levels in normal control group tissue and lymphoma tissue, and performed ROC curve analysis. We found AUC>0.9 and validated it in another dataset (AUC>0.8). GPX4 therefore has potential diagnostic value in clinical practice. Meanwhile, in order to better apply it to clinical practice (clinical tissue testing is invasive, while blood testing is more convenient), we analyzed GPX4's potential as a clinical diagnosis in peripheral blood and obtained results with AUC>0.9. GPX4 therefore has potential diagnostic value in clinical practice. Meanwhile, we found that GPX4's high expression had a better prognosis after chemotherapy compared to GPX4's low expression, and validated it in two other datasets. Meanwhile, we found that mutations in TP53 can lead to an rise in GPX4 expression in lymphoma patients. Compared with patients without mutations, patients with elevated GPX4 have a better prognosis, therefore GPX4's high expression has potential for good prognosis in clinical practice.

We preliminarily explored the mechanism by which GPX4's high expression leads to a good prognosis in patients through cell experiments, genes, and immune levels. Firstly, GPX4 blocks the cell cycle's S-phase and inhibits lymphocyte proliferation, while also inhibiting the expression of 16 key upregulated pathogenic genes in inhibiting CDCA7 gene's expression significantly and lymphoma. Literature reports suggest that CDCA7 plays an important role in lymphoma. DLBCL is a type of B-cell lymphoma, and GPX4 also inhibits B-cells' immune infiltration. Our findings may therefore provide new diagnostic's selection ideas and prognostic biomarkers for lymphoma, provide new insights into DLBCL with immune mechanisms, and help design effective DLBCL drug targets.

Inevitably, this study has several limitations. This study comes from public databases mainly and is retrospective. The number of clinical information datasets that is available for DLBCL patients is limited, so the clinical parameters that are analyzed in this study are not comprehensive. DLBCL patients need real clinical information to determine the value of GPX4. Secondly, although the function of GPX4 has been confirmed through two types of DLBCL cells, its upstream and downstream mechanisms are not clear, and more in-depth experiments are needed to study the mechanism, which will be the research we need in the future.

## Funding

This study was funded by Hangzhou science and technology Major Project with Grant Number: 202004A15, Hangzhou Medical health science and technology Major Project with Grant Number: Z20210039 and Zhejiang Province Traditional Chinese medicine science and technology project with Grant Number: 2023ZR122.

## Data availability statement

Gene expression datasets are publicly available (GEO, https://www.ncbi.nlm.nih.gov/geo).

## CRediT authorship contribution statement

**Can Chen:** Validation, Investigation, Data curation. **TongYu Li:** Investigation, Formal analysis, Data curation. **Yiwei Li:** Methodology, Investigation. **Zhenzhen Chen:** Methodology, Formal analysis. **Pengfei Shi:** Writing – original draft. **Yun Li:** Investigation, Project administration, Writing – original draft. **Shenxian Qian:** Funding acquisition, Software, Writing – review & editing.

## Declaration of competing interest

The authors declare that they have no known competing financial interests or personal relationships that could have appeared to influence the work reported in this paper.

## References

[1] Vodicka P., Klener P., Trneny M. (2022). Diffuse large B-cell lymphoma (DLBCL): early patient management and emerging treatment options. OncoTargets Ther..

[2] Shi H.Z., Pan Y.M., Xiang G.F. (2023). A novel NET-related gene signature for predicting DLBCL prognosis. J. Transl. Med..

[3] Zhang R.Y., Chen J.H., Wang S.Y. (2023). Ferroptosis in cancer progression. Cells.

[4] Xu T.Q., Liu Y.X., Zhao Z.W. (2023). Ferroptosis in cancer stem cells. Pathol. Res. Pract..

[5] M Weng J., Chen L., Liu H.C. (2022). Ferroptosis markers predict the survival, immune infiltration, and ibrutinib resistance of diffuse large B cell lymphoma. Inflammation.

[6] Liang D.G., Feng Y., Zandkarimi F. (2023). Ferroptosis surveillance independent of GPX4 and differentially regulated by sex hormones. Cell.

[7] Rochette L., Dogon G., Riga E. (2022). Lipid peroxidation and iron metabolism: two corner stones in the homeostasis control of ferroptosis. Int. J. Mol. Sci..

[8] Liu Y., Wan Y.C., Jiang Y. (2023). GPX4: the hub of lipid oxidation, ferroptosis, disease and treatment. Biochim. Biophys. Acta Rev. Canc.

[9] Ye Y.Z., Chen A., Li L. (2022). Repression of the antiporter SLC7A11/glutathione/glutathione peroxidase 4 axis drives ferroptosis of vascular smooth muscle cells to facilitate vascular calcification. Kidney Int..

[10] Ding Y.H., Chen X.P., Liu C. (2021). Identification of a small molecule as inducer of ferroptosis and apoptosis through ubiquitination of GPX4 in triple negative breast cancer cells. J. Hematol. Oncol..

[11] Miao Y., Chen Y.W., Xue F. (2022). Contribution of ferroptosis and GPX4's dual functions to osteoarthritis progression. EBioMedicine.

[12] Liu J., Kang R., Tang D.L. (2022). Signaling pathways and defense mechanisms of ferroptosis. FEBS J..

[13] Zhou C., Yu T., Zhu R. (2023). Timosaponin AIII promotes non-small-cell lung cancer ferroptosis through targeting and facilitating HSP90 mediated GPX4 ubiquitination and degradation. Int. J. Biol. Sci..

[14] Chen Y.L., Lin B.A., Yang S.Y. (2023). IRF1 suppresses colon cancer proliferation by reducing SPI1-mediated transcriptional activation of GPX4 and promoting ferroptosis. Exp. Cell Res..

[15] Cheng K., Huang Y.Q., Wang C.F. (2021). Inhibited ferroptosis in zebrafish liver cells (ZFL) by regulating keap1-nrf2-GPx4 and NF- κB-hepcidin Axis. Int. J. Mol. Sci..

[16] Dong S.Q., Lu Y.Y., Peng G.J. (2021). Furin inhibits epithelial cell injury and alleviates experimental colitis by activating the Nrf2-Gpx4 signaling pathway. Dig. Liver Dis..

[17] Wang D., Wei G.D., Ma J. (2021). Identification of the prognostic value of ferroptosis-related gene signature in breast cancer patients. BMC Cancer.

[18] Xie Y.C., Kang R., Klionsky D.J. (2023). GPX4 in cell death, autophagy, and disease. Autophagy.

[19] Taguchi T., Kurata M., Onishi I. (2021). SECISBP2 is a novel prognostic predictor that regulates selenoproteins in diffuse large B-cell lymphoma. Lab. Invest..

[20] Zhang Q.K., Zhu Z.S., Guan J.Q. (2022). Identification and assessment of necroptosis-related genes in clinical prognosis and immune cells in diffuse large B-cell lymphoma. Front. Oncol..

[21] Sheng L.X., Li T.Y., Li Y. (2023). Prognostic and immunological characterization of diffuse large B-cell lymphoma evaluated by co-stimulatory molecular-related features. Heliyon.

[22] Zhang B.X., Zhang T.Y., Zheng Z.W. (2023). Development and validation of a cuproptosis-associated prognostic model for diffuse large B-cell lymphoma. Front. Oncol..

[23] Wu J., Zhu H.Z., Zhang Q. (2023). Nomogram based on the systemic immune-inflammation index for predicting the prognosis of diffuse large B-cell lymphoma. Asia Pac. J. Clin. Oncol..

[24] Li C.C., Zhang Y., Xiao Y.Y. (2022). Identifying the effect of COVID-19 infection in multiple myeloma and diffuse large B-cell lymphoma patients using bioinformatics and system biology. Comput. Math. Methods Med..

[25] Feng L.L., Yan Q.Y., F L.X. (2023). Long non-coding RNA H19 recruits NFYB to activate MBTD1 and regulate doxorubicin resistance in lymphoma cells. Mol. Biotechnol..

[26] Martins E.P., Gonçalves C.S., Pojo M. (2022). Cadherin-3 is a novel oncogenic biomarker with prognostic value in glioblastoma. Mol. Oncol..

[27] Hua X.L., Ge S.D., Zhang J. (2021). A costimulatory molecule-related signature in regard to evaluation of prognosis and immune features for clear cell renal cell carcinoma. Cell Death Dis..

[28] Y Ma C., Wang D.D., Tian Z.F. (2023). USP13 deubiquitinates and stabilizes cyclin D1 to promote gastric cancer cell cycle progression and cell proliferation. Oncogene.

[29] Shi M.Y., Wang Y.R., Shi Y. (2023). SETDB1-mediated CD147-K71 di-methylation promotes cell apoptosis in non-small cell lung cancer. Genes Dis.

[30] Cui Y.Y., Leng C.S. (2023). A glycolysis-related gene signatures in diffuse large B-Cell lymphoma predicts prognosis and tumor immune microenvironment. Front. Cell Dev. Biol..

[31] Li C.L., Zhang J.L., Bi Y.W. (2023). Unveiling the prognostic significance of SOX5 in esophageal squamous cell carcinoma: a comprehensive bioinformatic and experimental analysis. Aging (Albany NY).

[32] Yan X., Lai B.B., Zhou X.Y. (2022). The differential expression of CD47 may be related to the pathogenesis from myelodysplastic syndromes to acute myeloid leukemia. Front. Oncol..

[33] Wang P., Lu Y.Q. (2022). Ferroptosis: a critical moderator in the life cycle of immune cells. Front. Immunol..

[34] Li S.B., He Y.P., Chen K.X. (2021). RSL3 drives ferroptosis through NF-κB pathway activation and GPX4 depletion in glioblastoma. Oxid. Med. Cell. Longev..

[35] Zheng C.X., Wang C., Sun D. (2023). Structure-activity relationship study of RSL3-based GPX4 degraders and its potential noncovalent optimization. Eur. J. Med. Chem..

[36] Wang Y.H., Zhao Y., Zhang Z.Y. (2023). High expression of CDCA7 in the prognosis of glioma and its relationship with ferroptosis and immunity. Genes.

[37] Li X.Y., Dong H., Zheng Y.F. (2023). AKAP12 inhibits esophageal squamous carcinoma cell proliferation, migration, and cell cycle via the PI3K/AKT signaling pathway. Mol. Cell. Probes.

[38] Chen J.H., Jin H.W., Zhou H. (2023). Research into the characteristic molecules significantly affecting liver cancer immunotherapy. Front. Immunol..

