# Supplementary figures and images for: GPX4 is a potential diagnostic and therapeutic biomarker associated with diffuse large B lymphoma cell proliferation and B cell immune infiltration
Source: Heliyon. 2024 Jan 26;10(3):e24857. doi: 10.1016/j.heliyon.2024.e24857 (PMC10850411; doi:10.1016/j.heliyon.2024.e24857)

A

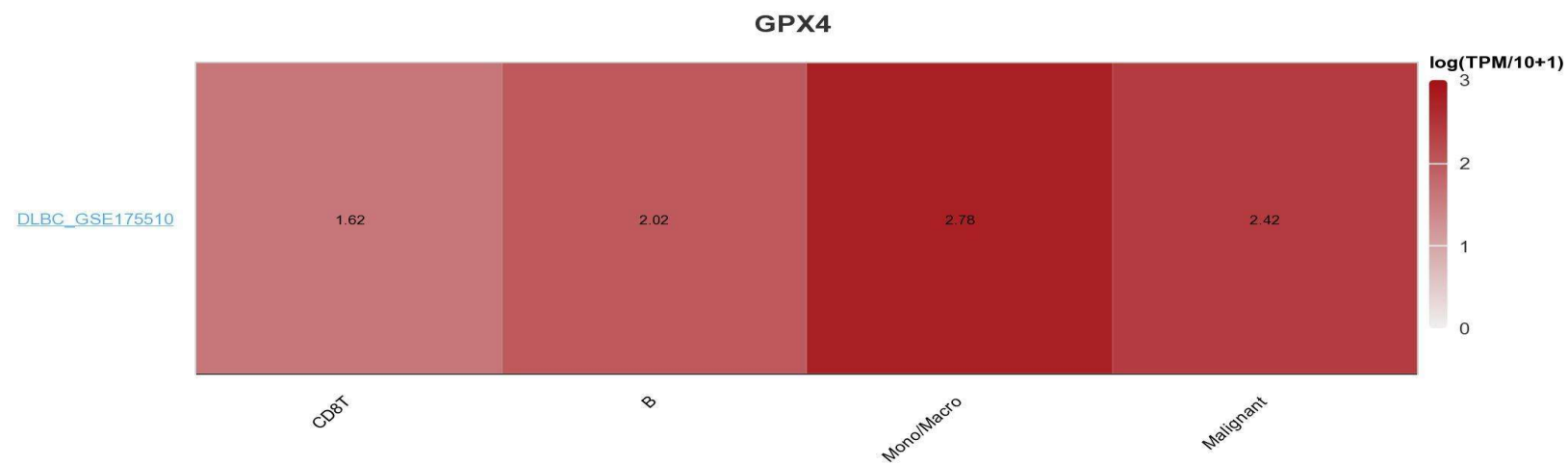

B

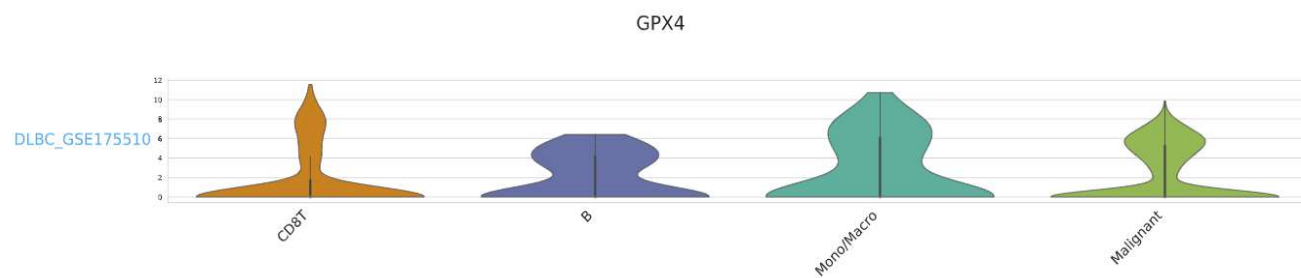

C

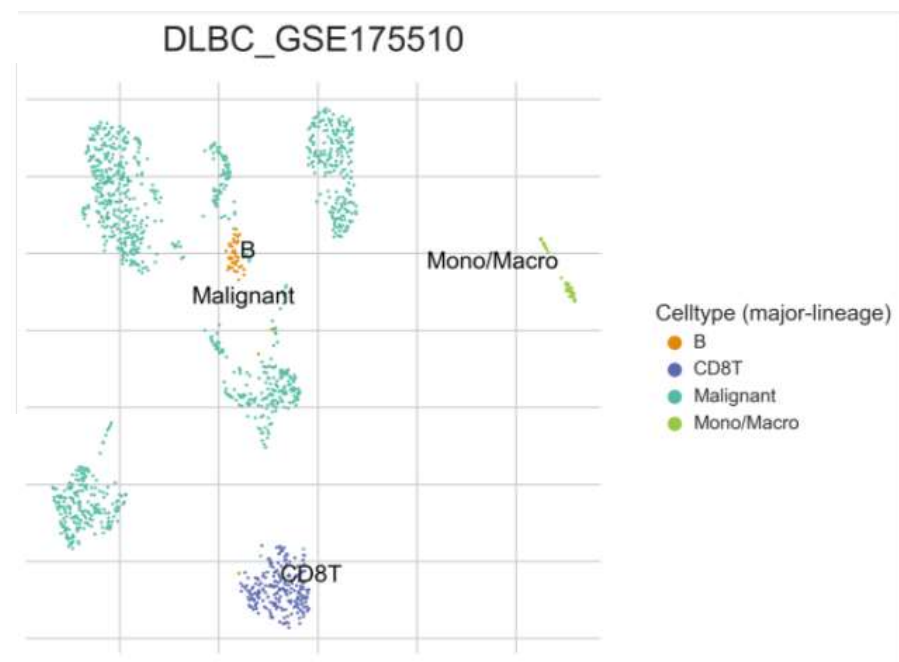

Supplement: Multimedia component 1 [file mmc1.pdf]

**A** Kruskal–Wallis test  $p=2.8e-20$

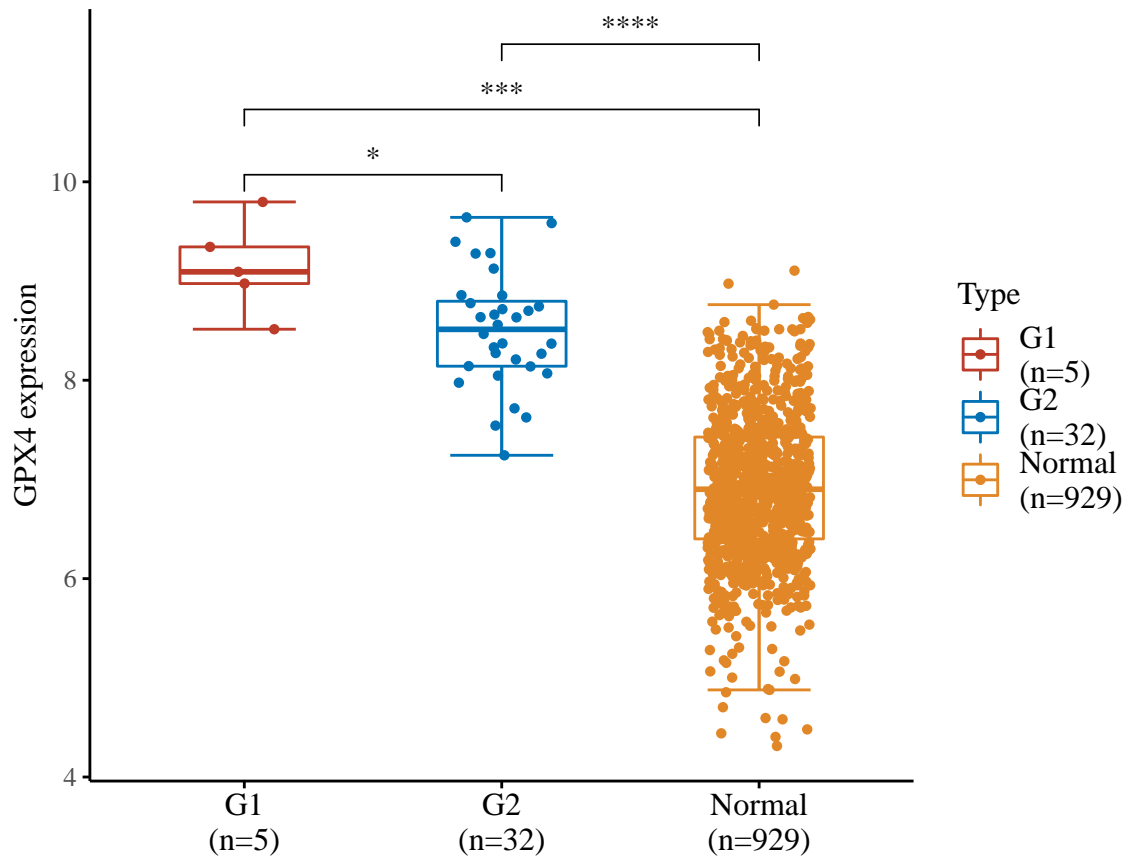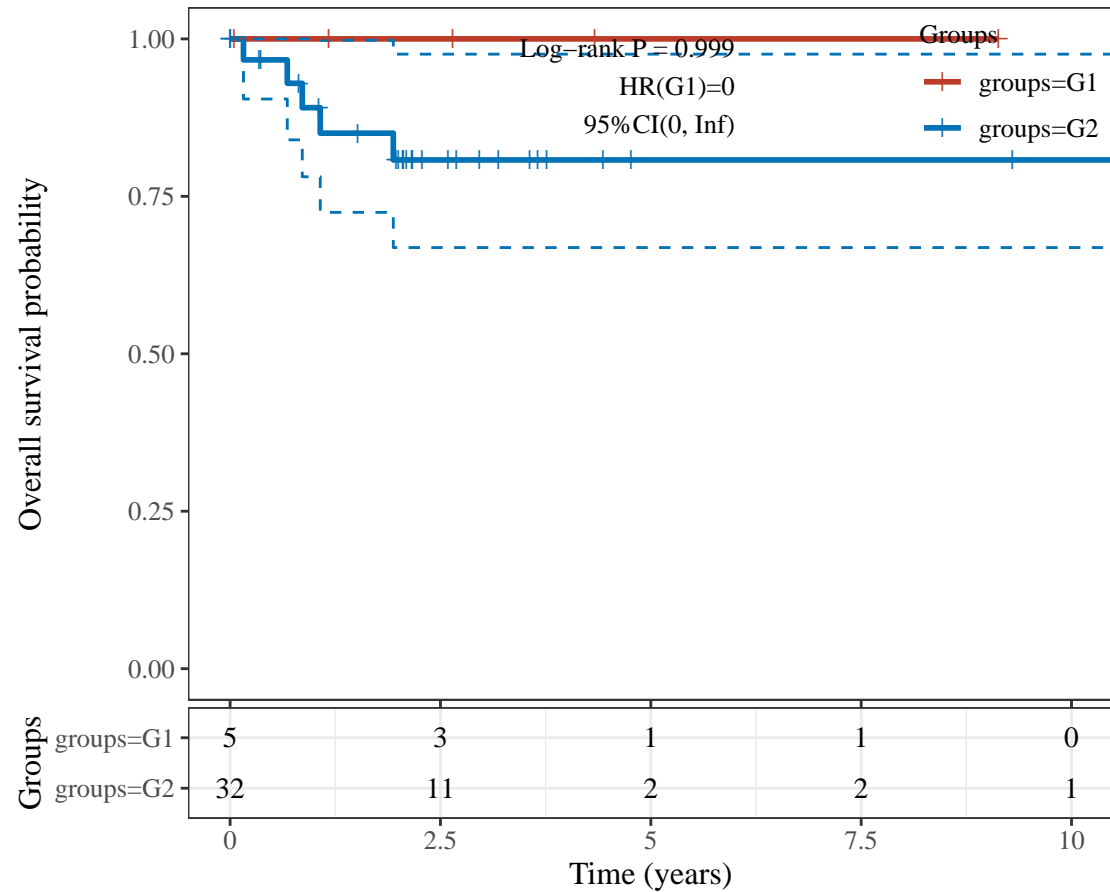

Supplement: Multimedia component 2 [file mmc2.pdf]

NC

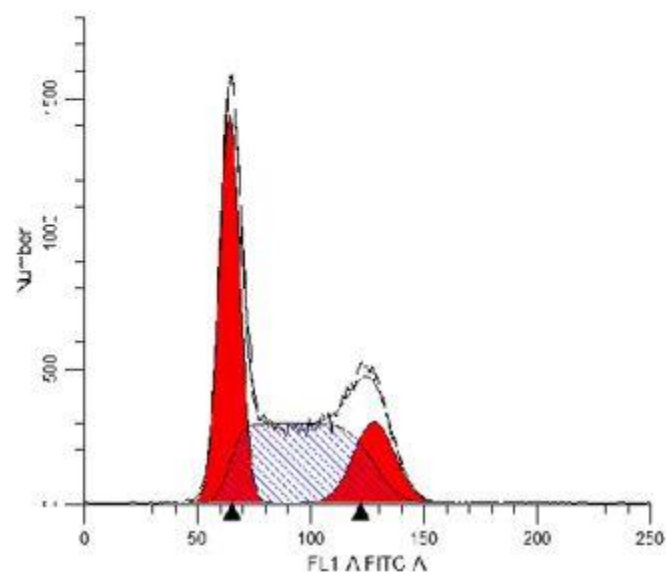

Diploid: 100.00 %  
 Dip G1: 39.19 % at 63.95  
 Dip G2: 16.34 % at 127.89  
 Dip S: 44.47 % G2/G1: 2.00  
 ◇ %CV: 7.16

过表达

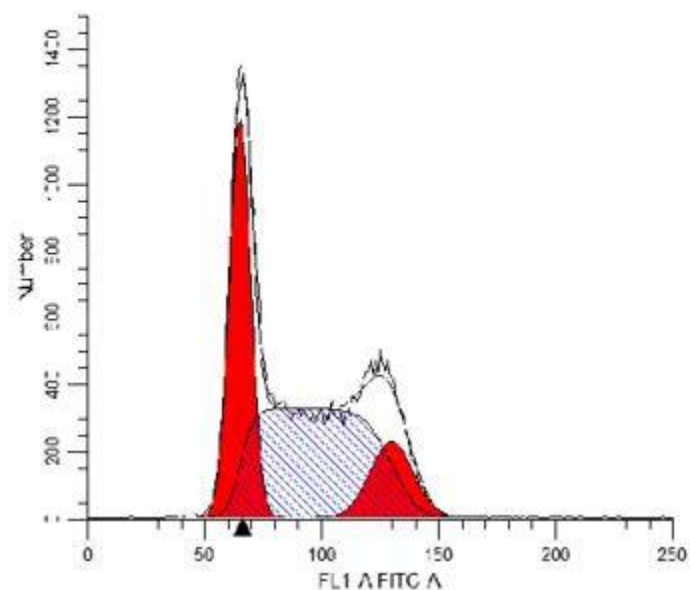

Diploid: 100.00 %  
 Dip G1: 34.29 % at 64.90  
 Dip G2: 13.17 % at 129.80  
 Dip S: 52.55 % G2/G1: 2.00  
 ◇ %CV: 7.06

Supplement: Multimedia component 3 [file mmc3.pdf]

A

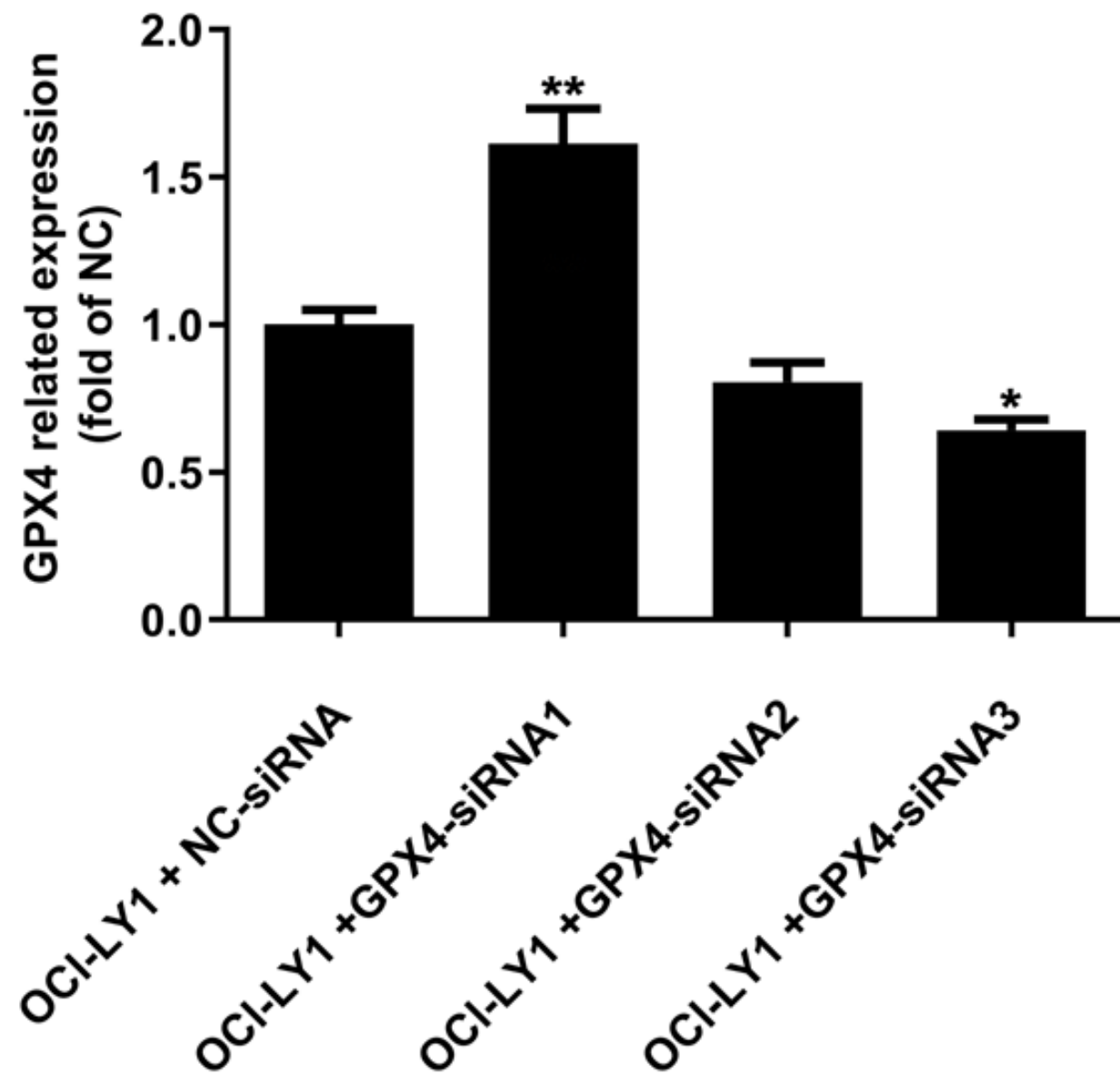

Supplement: Multimedia component 4 [file mmc4.pdf]

**A**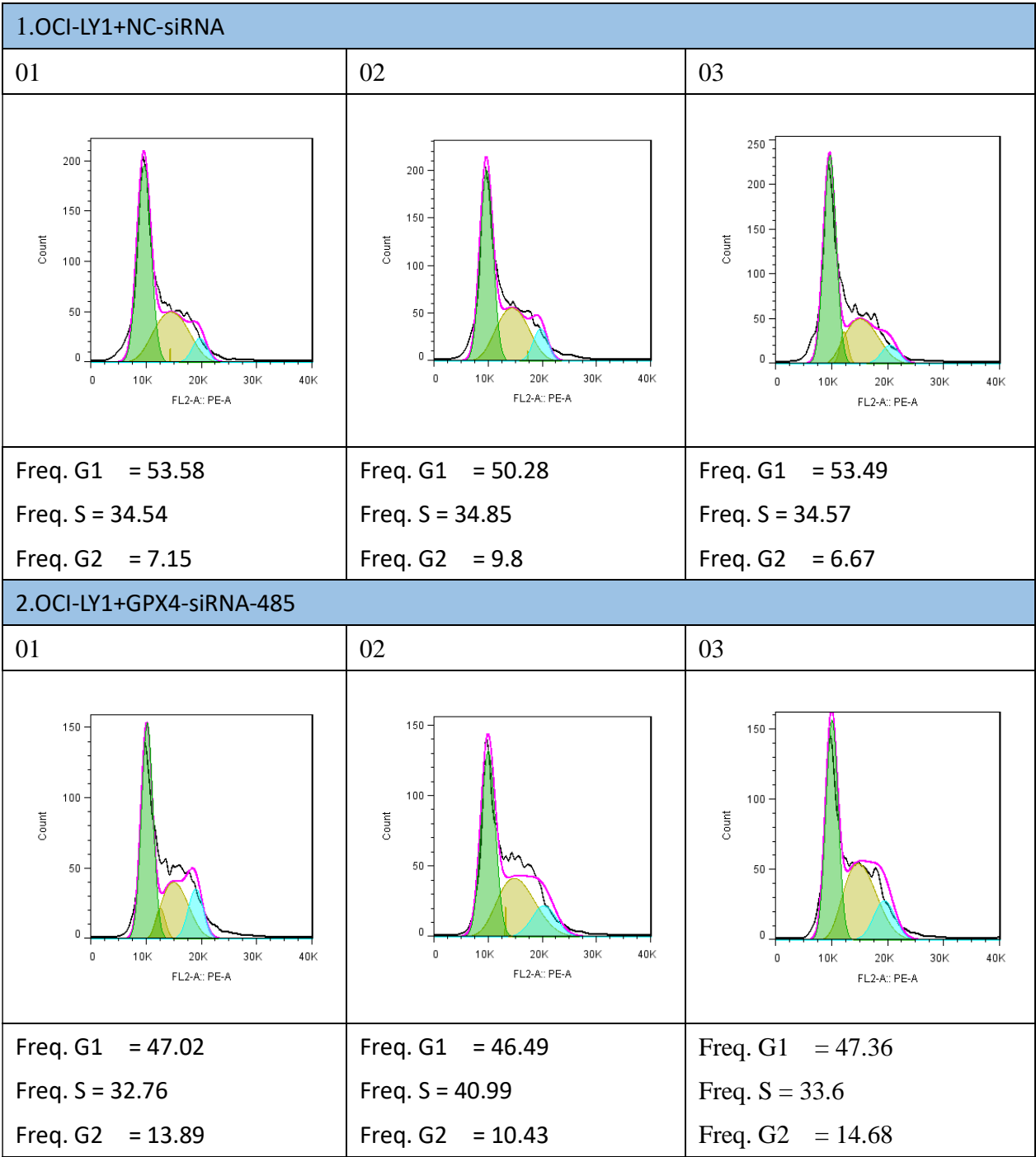**B**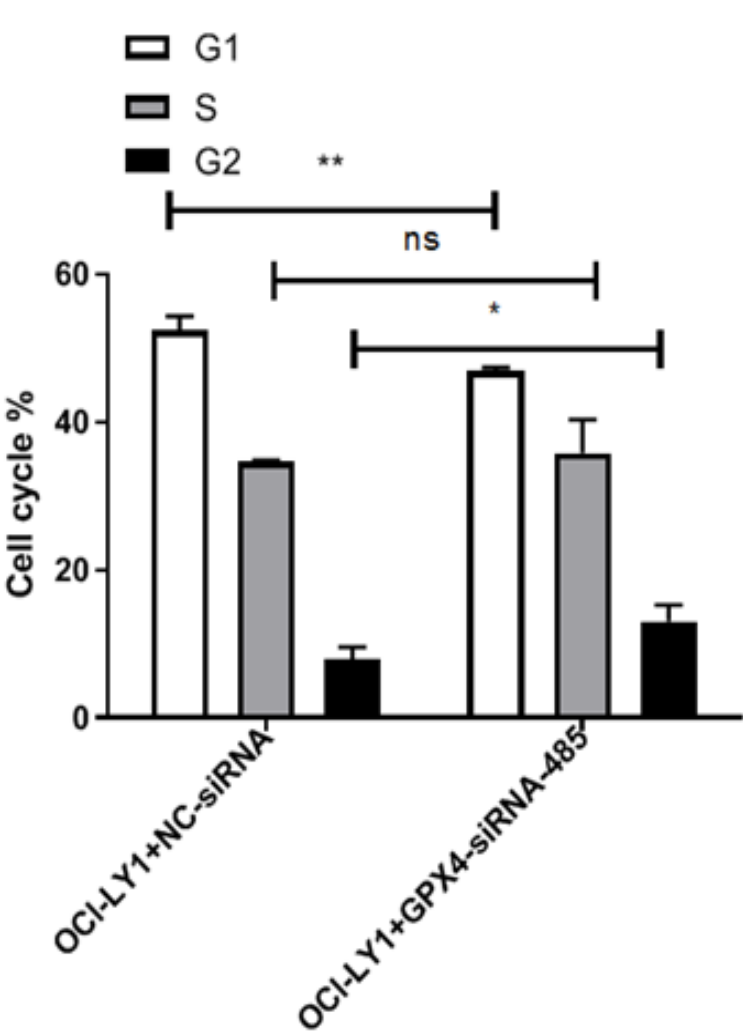

Supplement: Multimedia component 5 [file mmc5.pdf]

A

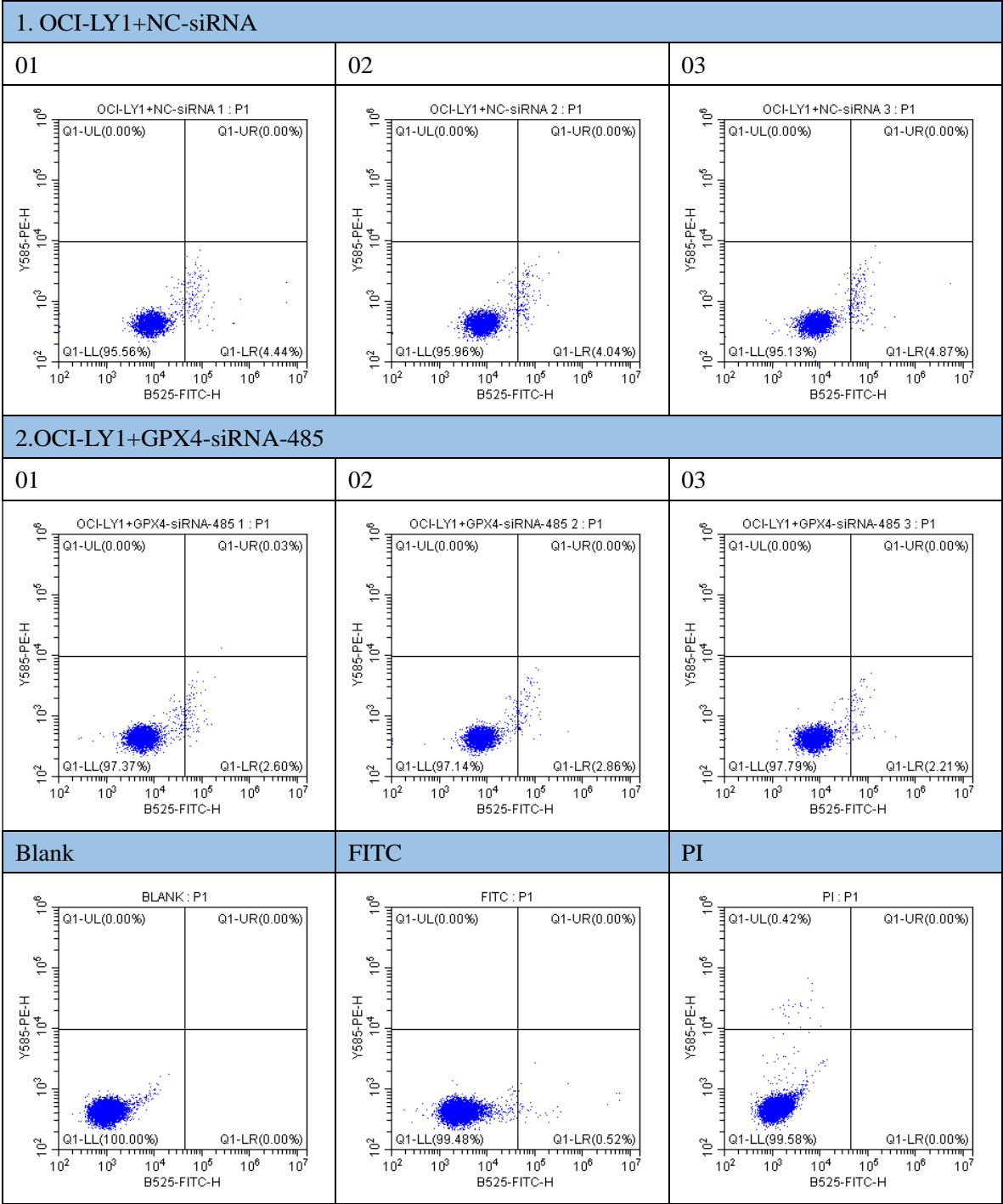

B

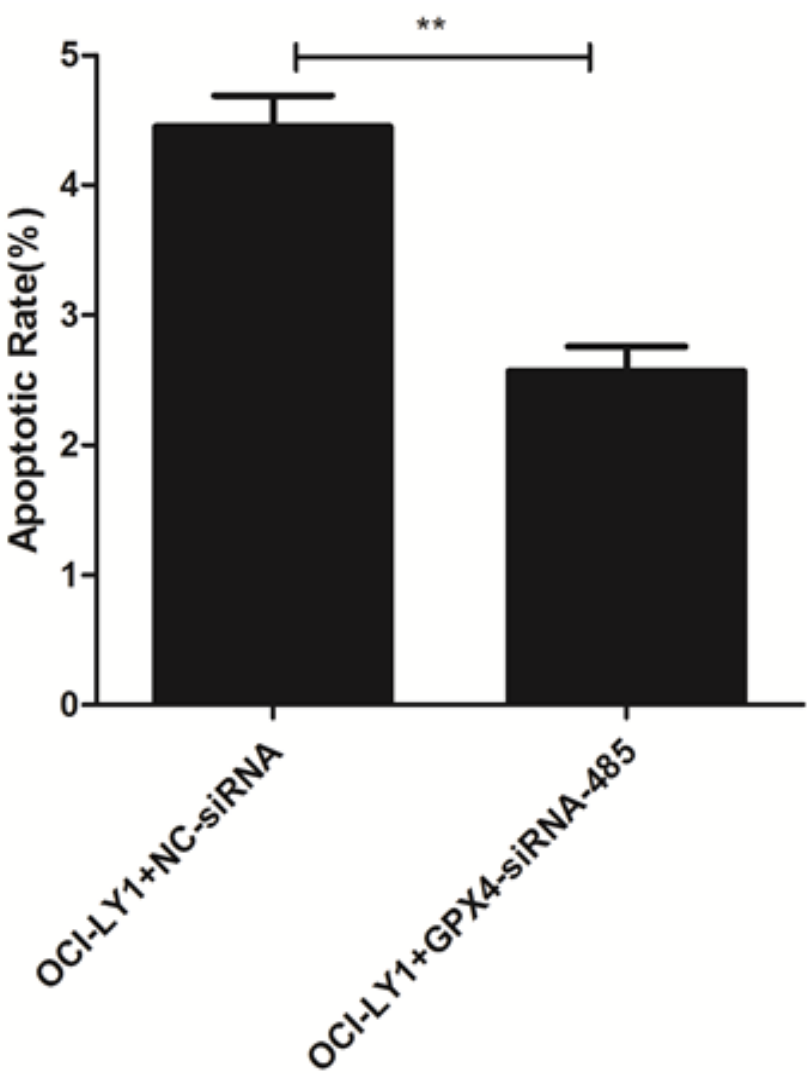

Supplement: Multimedia component 6 [file mmc6.pdf]
