# Supplementary material for: GPX4 is a potential diagnostic and therapeutic biomarker associated with diffuse large B lymphoma cell proliferation and B cell immune infiltration
Source: Heliyon. 2024 Jan 26;10(3):e24857. doi: 10.1016/j.heliyon.2024.e24857 (PMC10850411; doi:10.1016/j.heliyon.2024.e24857)
Supplement: Multimedia component 7 [file mmc7.docx]

**Figure S1 According to the DLBCL dataset GSE175510**

we found that GPX4 is highly expressed in four types of immune cells, thus GPX4 has potential functions in the pathological and immune mechanisms of DLBCL(A-C).

.

**Figure S2 Differences in GPX4 expression levels between TP53 mutant and TP53 wild-type diffuse large B-cell lymphoma from the TCGA dataset (shown on the left) Kaplan Meier curve (shown on the right),** **G1 represents DLBCL samples of TP53 mutant type, G2 represents samples of TP53 wild-type, and Normal represents samples of healthy control group**

According to the graph, it can be found that there is a significant difference in GPX4 expression between the TP53 mutant and the TP53 wild-type and Norma groups (Kruskal−Wallis test p=2.8e−20). The TP53 mutant has the highest GPX4 expression (shown on the left) and the best prognosis (shown on the right, red representing the high risk scores and blue representing the low risk scores) (A).

**Figure S3 The Functional Role of High Expression GPX4 in DLBCL Cells**

The NC group represents the empty group (shown on the left), while the overexpression group represents the GPX4 high expression transfected cell group (shown on the right). The experiment of overexpressing GPX4 transfected cells found that high expression of GPX4 promoted the G1 synthesis phase, but blocked cell synthesis in the S phase, thereby reducing cell reduction in the G2 synthesis phase. Therefore, high expression of GPX4 accelerated cell arrest in the S phase, thereby inhibiting the proliferation of DLBCL cells(A).

**Figure S4 Construction of Cell Model for GPX4 Interference**

The qPCR results showed that compared with the control group, the cells transfected with OCI-LY1+GPX4 -siRNA3 had the most significant interference effect on GPX4 (A).

**Figure S5 DLBCL Cell Cycle Experiment.**

The cell cycle results showed that Compared with group OCI-LY1+NC-siRNA cells, group OCI-LY1+GPX4-siRNA-485 showed a decrease in G1 phase cells, no significant changes in S phase cells, and an increase in G2 phase cells (A-B).

**Figure S6 DLBCL cell apoptosis experiment.**

The results of cell apoptosis showed that the OCI-LY1+GPX4-siRNA-485 group showed a significant decrease in the apoptosis rate compared to the OCI-LY1+NC-siRNA group cells (A-B).
